# Longitudinal biological aging trajectories and incident urolithiasis: a causal mediation analysis role of metabolic dysfunction in a large Chinese cohort

**DOI:** 10.3389/fendo.2026.1824716

**Published:** 2026-04-17

**Authors:** Jialong Wang, Xingzhou Guo, Xingyin Wang, Zeyuan Song, Sishuai Mao, Yifan Li

**Affiliations:** Department of Urology, Affiliated Hospital of Yangzhou University, Yangzhou, China

**Keywords:** biological aging, geroscience, Klemera-Doubal, mediation analysis, trajectory analysis, urolithiasis

## Abstract

**Background:**

Biological aging varies significantly between individuals, yet how its longitudinal dynamics influence urolithiasis risk remains poorly understood. This investigation sought to delineate distinct biological aging trajectories and quantify the mediating role of metabolic dysfunction in incident stone formation.

**Methods:**

We analyzed longitudinal physiological data from 27, 948 participants who underwent routine health screenings at the Affiliated Hospital of Yangzhou University (HMC-AHYU) between 2022 and 2024. The kml3d unsupervised machine learning algorithm was employed to identify latent trajectories of biological aging based on the Klemera-Doubal method (KDM) and Homeostatic Dysregulation (HD). We used Cox proportional hazards models and causal mediation analyses to evaluate the association between aging patterns and incident urolithiasis.

**Results:**

Trajectory modeling delineated four distinct patterns of biological aging: Stable Low-Risk (Class A), Stable Moderate-Risk (Class B), Remissive High-Risk (Class C), and Progressive High-Risk (Class D). Compared to the Stable Low-Risk group, the Progressive High-Risk group was associated with an increased risk of lithogenesis in the unadjusted model (HR = 1.21, 95% CI: 1.00–1.45, P = 0.047), which was fully attenuated after adjusting for metabolic factors (HR = 1.14, 95% CI: 0.95–1.37, P = 0.170). Causal mediation analysis confirmed that this association was predominantly driven by BMI (22.9%, P<0.001) and hypertension (10.4%, P = 0.032). Conversely, the Remissive High-Risk group maintained a significantly lower risk across all models (HR = 0.87, 95% CI: 0.76–0.99, P = 0.041).

**Conclusions:**

Longitudinal biological aging trajectories are robust early predictors of urolithiasis. While progressive aging increases risk via metabolic factors (obesity and hypertension), the remissive trajectory suggests that physiological recovery is possible. Targeting these metabolic mediators may offer an effective strategy for stone prevention.

## 1 Introduction

Urolithiasis is a major healthcare burden, particularly in China, where rapid economic transitions have coincided with rising rates (1–3). Prevalence is estimated between 6.4% and 8.1%, with higher rates in the south (3, 4). Given that stone recurrence rates approach 50% within five years (5, 6), and standard risk factors like obesity often fail to capture the full picture (7–9), identifying upstream, subclinical drivers is essential.

The Geroscience hypothesis holds that fundamental biological aging processes drive most chronic pathologies (10). Because relying solely on chronological age fails to accurately reflect internal physiological deterioration, metrics such as the Klemera-Doubal Method (KDM) and Homeostatic Dysregulation (HD) are used to quantify systemic dysregulation (11, 12). While accelerated biological aging predicts cardiovascular outcomes (13, 14), its role in urolithiasis remains unclear.

Crucially, most studies measure biological age only once. This cross-sectional approach misses the dynamic nature of aging (15). Longitudinal trajectories, which track the speed and direction of changes, predict outcomes better than snapshots (16). Distinguishing between stable high-risk states and rapidly accelerating phenotypes is vital for precise risk stratification (17).

In this study, we analyzed longitudinal data collected from 2022 to 2024 at the Affiliated Hospital of Yangzhou University (HMC-AHYU). We used the unsupervised kml3d algorithm to identify joint latent trajectories of KDM and HD. We tested whether distinct aging patterns predict incident urolithiasis and measured how much modifiable metabolic factors mediate this risk.

## 2 Materials and methods

### 2.1 Ethics approval and consent to participate

This study was approved by the Institutional Review Board of the Affiliated Hospital of Yangzhou University (Approval No. 2025-YKL06-K05) and conducted in accordance with the Declaration of Helsinki. Given the retrospective nature of the study, the requirement for written informed consent was waived by the Ethics Committee. To ensure privacy, all participant data were de-identified and analyzed anonymously.

### 2.2 Study population and design

The present study is predicated on data from adults in HMC-AHYU from 2022 to 2024. HMC-AHYU data, collected by certified staff after a 12-hour fast, comprised demographics, medical history, standard dry-chemistry laboratory tests, and intermediate-rank sonologists’ ultrasound findings. The institutional ethics committee granted a waiver of informed consent. A total of 39, 357 participants were initially enrolled from the HMC-AHYU cohort. Exclusion criteria included a history of kidney transplantation or nephrectomy (n = 9), without ultrasound of the liver and kidney (n = 75), and incomplete data on specific biomarkers required for biological age calculation, including albumin (Alb), creatinine, fasting glucose, blood urea nitrogen (BUN), lymphocyte percentage, mean cell volume (MCV), red cell distribution width (RDW), and white blood cell count (WBC) (n = 11, 325). All remaining variables were complete. The final analysis comprised 27, 948 participants (**Supplementary Figure 1**). The reporting of this study follows the STROBE reporting guideline for cohort studies.

### 2.3 Exposure

The primary exposure of interest was the joint longitudinal trajectory of biological aging, characterized by two distinct metrics: KDM biological age acceleration (KDM-advance) and log-transformed HD (HD-log). Both metrics were computed from a panel of eight routine blood and biochemical markers, encompassing renal function (creatinine, BUN), glycemic status (fasting glucose), and hematological indices (Alb, lymphocyte percentage, MCV, RDW, and WBC). KDM-advance represents the deviation of biological age from chronological age, calculated as the residual from the regression of biomarkers on age in the NHANES III reference population (11). HD-log was computed as the log-transformed Mahalanobis distance, summarizing multidimensional divergence from the reference centroid (12). The mathematical formulations for these metrics are presented below:


$$BA_{KDM}=\frac{\left(\sum_{j=1}^{m}(\frac{x_{j}-\;q_{j}}{k_{j}})\cdot (\frac{k_{j}^{2}}{s_{j}^{2}})+\frac{CA}{\sigma^{2}}\right)}{\left(\sum_{j=1}^{m}(\frac{k_{j}^{2}}{s_{j}^{2}})+\frac{1}{\sigma^{2}}\right)}$$

$$KDM_{Advance}=\;BA_{KDM}-\;CA$$

$$HD\;=\;D_{M}(x)=\;\sqrt{{\left(x\;-\;\mu \right)}^{T}S^{-1}(x\;-\;\mu )}$$

$$HD_{Log}=\;ln(HD)$$

Where *x_j_* represents the observed value of biomarker *j* for the individual; *k_j_*, *q_j_*, and *s_j_* denote the slope, intercept, and root mean square error, respectively, derived from the regression of biomarker *j* on chronological age in the healthy reference population; *CA* is the individual’s chronological age; and *σ*^2^ is the variance of chronological age within the reference population. Regarding the HD algorithm, *x* represents the vector of biomarker values; *μ* denotes the mean vector of the reference population; *S*^–1^ is the inverse variance-covariance matrix; and *T* indicates the vector transpose.

We used the kml3d algorithm to identify joint trajectories of biological aging. This method is specifically designed for clustering multivariate longitudinal data. We determined the optimal number of clusters (k) by testing values from 2 to 6. Model selection was based on the Calinski-Harabasz (CH) index, the Akaike Information Criterion (AIC), and the Bayesian Information Criterion (BIC). We also assessed classification accuracy using the Average Posterior Probability (AvePP), requiring a threshold of >0.70 for reliable separation. To ensure statistical stability, we required the smallest latent class to include at least 5% of the study population. The final k was selected based on clinical interpretability rather than statistical metrics alone.

### 2.4 Urolithiasis

In this study, urolithiasis was diagnosed by B-mode ultrasonography using a 3.5–5 MHz convex abdominal probe (LOGIQ E9, GE, USA). While non-contrast CT is the reference standard for stone detection, ultrasonography remains the primary screening modality in large-scale longitudinal cohorts due to its non-invasive nature and the absence of ionizing radiation. All scans and interpretations were performed by experienced sonographers (> 5 years of practice), with findings integrated into the clinical context and independently reviewed by a second sonographer to minimize inter-observer variability. Kidney stones were defined as echogenic foci ≥ 2 mm with posterior acoustic shadowing. This threshold was predefined to maximize diagnostic specificity, acknowledging that microlithiasis (< 2 mm) may be underdetected, potentially yielding a conservative estimate of the true association.

### 2.5 Definitions of covariates

Covariates included age, gender, body mass index (BMI), systolic blood pressure (SBP), diastolic blood pressure (DBP), hypertension, and diabetes. All blood samples were collected in the morning following an overnight fast of at least 12 hours. The diagnostic criteria for hypertension involved recorded blood pressure readings exceeding 140/90 mmHg, a verified medical history of the condition, or the active use of antihypertensive pharmacotherapy. Diabetes mellitus was defined based on a self-reported physician diagnosis, or the current use of insulin or oral antidiabetic agents.

### 2.6 Statistical analysis

Continuous variables were assessed for normality using the Kolmogorov-Smirnov test. Given the large sample size, we relied on the Central Limit Theorem to assume the sampling distribution of the means approximated normality. We present continuous variables as mean (SD) and compared them using one-way ANOVA followed by Tukey-Kramer *post hoc* tests. Categorical variables are reported as frequencies (%) and were compared using the chi-square test. No missing values were present in the covariates used for the multivariable models.

To identify baseline predictors of trajectory group membership, we used multinomial logistic regression. The Stable Low-Risk group (Class A) served as the reference category. We calculated odds ratios (ORs) and 95% confidence intervals (CIs) to evaluate associations with the likelihood of being assigned to the Stable Moderate-Risk (Class B), Remissive High-Risk (Class C), or Progressive High-Risk (Class D) groups.

We fitted Cox proportional hazards regression models to estimate hazard ratios (HRs) and 95% CIs for incident urolithiasis. The proportional hazards assumption was verified using Schoenfeld residuals. We constructed three models: Model 1 was unadjusted; Model 2 adjusted for age, gender, and BMI; and Model 3 was fully adjusted for age, gender, BMI, SBP, DBP, hypertension, and diabetes. Multicollinearity was assessed using the variance inflation factor.

To ensure the robustness of our findings and minimize reverse causality, we conducted sensitivity analyses by excluding participants with baseline hypertension or diabetes, thereby restricting the cohort to a metabolically healthy population. We also performed subgroup analyses and tests for interaction to evaluate potential effect modification by age, gender, BMI, and metabolic comorbidities.

To directly test the mediating role of metabolic dysfunction, a formal causal mediation analysis was performed based on the counterfactual framework. We decomposed the total effect of aging trajectories into the Average Direct Effect (ADE) and the Average Causal Mediation Effect (ACME) using the mediation package in R. We validated the significance of indirect effects using bias-corrected bootstrapping with 1000 resamples (18). The proportion of the total effect mediated was calculated as the indirect effect divided by the total effect. All statistical analyses were conducted using R software (version 4.5.1), and a two-sided P value < 0.05 was considered statistically significant.

## 3 Results

### 3.1 Analysis of joint trajectories and baseline characteristics

We fitted joint trajectory models for 2–6 clusters and assessed their performance using various model fit indices (**Supplementary Table 1**). We determined the optimal class number by balancing statistical parsimony with clinical resolution. While the CH index favored fewer clusters, AIC and BIC values indicated a better fit for the four-class solution over two- or three-class models. This model also showed high classification certainty, with AvePP exceeding 0.90 for all subgroups (**Supplementary Table 2**). Regarding distribution, the solution met the requirement that all subgroups constitute at least 5% of the population, with the smallest group accounting for 7.23%. Given these metrics, we proceeded with the four-class solution for subsequent analyses.

**Figure 1:**
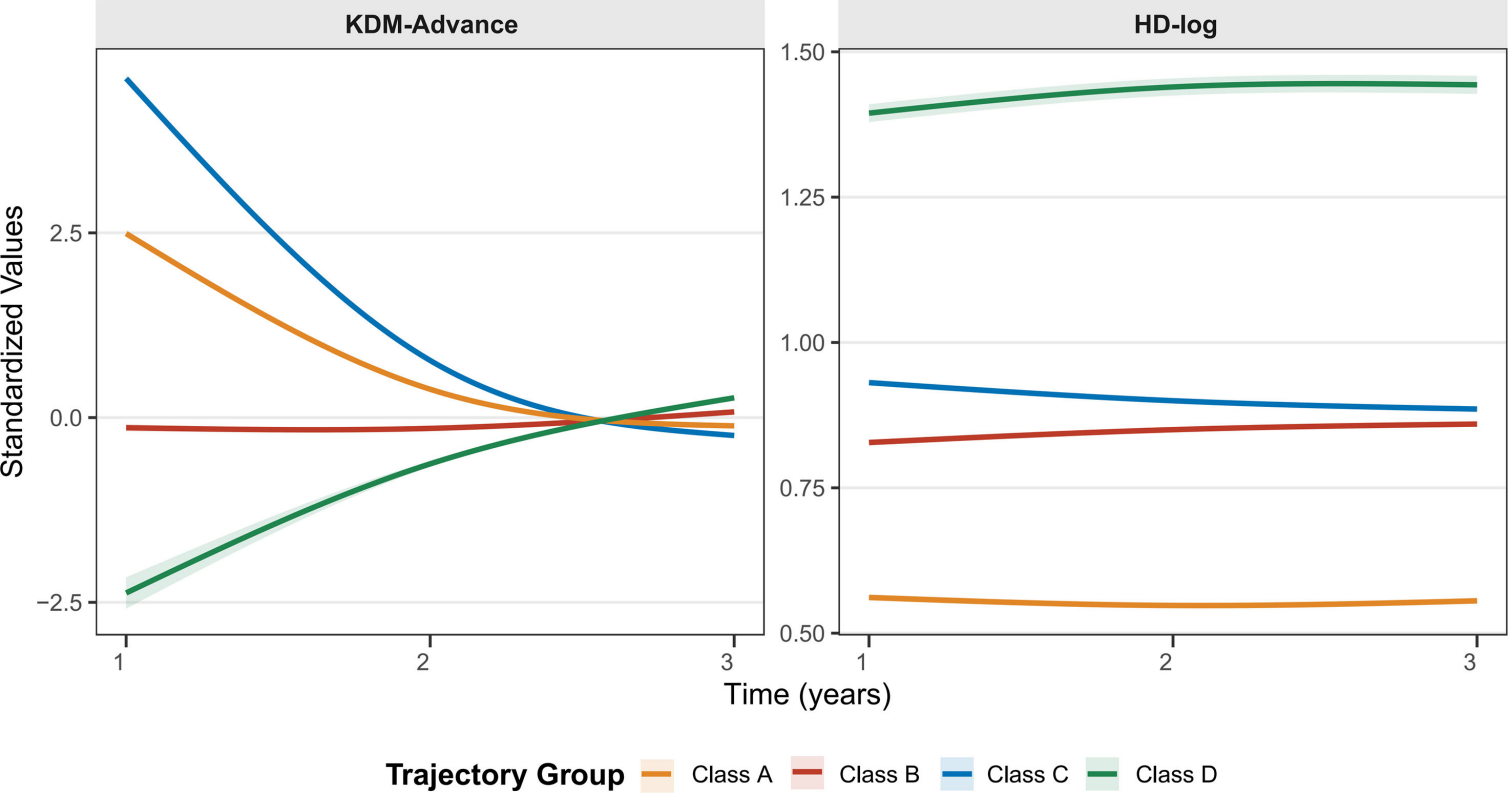
Longitudinal trajectories of biological aging identified by unsupervised clustering. Joint trajectories of KDM-advance (left) and HD-log (right) over 3 years revealed four distinct groups: Class A (Stable Low-Risk, Reference), Class B (Stable Moderate-Risk), Class C (Remissive High-Risk), and Class D (Progressive High-Risk). Standardized values (y-axis) are Z-score transformed; 0 represents the cohort mean at baseline, with higher values indicating more advanced biological aging. Shaded areas denote 95% CIs. CI, confidence interval; KDM-advance, Klemera-Doubal Method biological age acceleration; and HD-log, log-transformed Homeostatic Dysregulation.

Four distinct physiological aging trajectories were identified via the kml3d algorithm (**Figure 1**). The Stable Moderate-Risk group remained steady, with KDM-advance fluctuating near zero and intermediate homeostatic dysregulation. The Stable Low-Risk group carried the lowest systemic burden, maintaining minimal HD-log levels throughout. This pattern suggested optimal physiological stability. The Remissive High-Risk group began with elevated biological age acceleration but saw values decline sharply toward baseline. In contrast, the Progressive High-Risk group showed the most adverse phenotype, defined by an escalating trajectory of biological age acceleration and persistently high homeostatic dysregulation.

The study included 27, 948 participants, with baseline characteristics and urolithiasis distribution summarized in **Table 1**. Overall urolithiasis prevalence was 7.49% (2, 094 cases). Characteristics and stone distribution differed significantly by trajectory. The Progressive High-Risk group had the highest stone incidence (9.72%), followed by the Stable Low-Risk, Stable Moderate-Risk, and Remissive High-Risk groups (P < 0.001). Notably, the Progressive High-Risk group showed a unique pre-clinical metabolic profile. Despite being the youngest participants with the lowest rates of diagnosed hypertension and diabetes, they suffered the most severe physiological dysregulation. This group had the highest BMI, SBP, and HD-log compared to all other groups (all P < 0.001).

**Table 1:** Baseline characteristics of the study population stratified by biological aging trajectories.

Variables	Stable low-risk (Class A)	Stable moderate-risk (Class B)	Remissive high-risk (Class C)	Progressive high-risk (Class D)	P value
N	7633	9687	8607	2021	
Age (years)	51.21 ± 14.91	49.72 ± 14.30	52.55 ± 14.81	49.61 ± 13.46	<0.001
Gender (%)					0.079
Male	5211 (68.27%)	6589 (68.02%)	5995 (69.65%)	1402 (69.37%)	
Female	2422 (31.73%)	3098 (31.98%)	2612 (30.35%)	619 (30.63%)	
Hypertension (%)					<0.001
No	4849 (63.53%)	6393 (66.00%)	5204 (60.46%)	1329 (65.76%)	
Yes	2784 (36.47%)	3294 (34.00%)	3403 (39.54%)	692 (34.24%)	
Diabetes (%)					<0.001
No	6900 (90.40%)	8886 (91.73%)	7745 (89.98%)	1862 (92.13%)	
Yes	733 (9.60%)	801 (8.27%)	862 (10.02%)	159 (7.87%)	
Alb (g/dL)	4.47 ± 0.25	4.48 ± 0.25	4.46 ± 0.25	4.47 ± 0.25	0.002
Creatinine (mg/dL)	0.78 ± 0.18	0.78 ± 0.19	0.79 ± 0.18	0.79 ± 0.23	0.011
BUN (mg/dL)	14.62 ± 3.72	14.41 ± 3.64	14.65 ± 3.74	14.44 ± 3.52	<0.001
WBC(*10^9^/L)	6.38 ± 1.57	6.39 ± 1.54	6.39 ± 1.85	6.42 ± 1.79	0.793
Lymphocyte percentage (%)	34.60 ± 7.65	34.88 ± 7.60	34.68 ± 7.67	34.70 ± 7.48	0.102
MCV (fL)	91.66 ± 4.92	91.64 ± 4.86	91.90 ± 4.74	91.62 ± 5.02	0.001
RDW (%)	12.93 ± 0.96	12.90 ± 0.93	12.91 ± 0.86	12.90 ± 0.93	0.202
Fasting glucose(mg/dL)	100.46 ± 25.28	99.31 ± 23.54	100.66 ± 24.02	99.00 ± 21.07	<0.001
BMI (kg/m2)	24.58 ± 3.23	23.69 ± 3.26	24.94 ± 3.43	24.90 ± 3.68	<0.001
SBP (mmHg)	125.79 ± 17.65	128.61 ± 20.06	126.85 ± 17.35	134.02 ± 20.70	<0.001
DBP (mmHg)	79.67 ± 11.52	78.88 ± 11.58	80.11 ± 11.51	81.29 ± 12.30	<0.001
HD-log	0.53 ± 0.24	0.92 ± 0.21	0.84 ± 0.24	1.46 ± 0.34	<0.001
KDM-advance(years)	2.41 ± 1.89	4.91 ± 1.93	-0.74 ± 1.90	-2.84 ± 5.03	<0.001
Incident urolithiasis (%)					0.009
No	7201 (94.34%)	9190 (94.87%)	8071 (93.77%)	1894 (93.72%)	
Yes	432 (5.66%)	497 (5.13%)	536 (6.23%)	127 (6.28%)	

Alb, Albumin; BUN, blood urea nitrogen; MCV, mean cell volume; RDW, red cell distribution width; WBC, white blood cell count; BMI, body mass index; SBP, systolic blood pressure; DBP, diastolic blood pressure; KDM-advance, Klemera-Doubal Method biological age acceleration; and HD-log, log-transformed Homeostatic Dysregulation.

### 3.2 Determinants of trajectory group membership

Multinomial logistic regression identified BMI as a robust predictor of trajectory membership (**Table 2**). To enhance clinical interpretability, we scaled the odds ratios to a 5-unit increment in BMI. Every 5 kg/m² increase in BMI was associated with a 16% higher likelihood of belonging to the Progressive High-Risk group (OR = 1.16; 95% CI: 1.05–1.28; P < 0.001). Similarly, age was modeled per 5-year increment, revealing that younger participants were more likely to be assigned to the Progressive High-Risk group (OR = 0.81 per 5 years; P < 0.001).

**Table 2:** Baseline predictors of biological aging trajectory membership: a multinomial logistic regression analysis.

Variables	Stable moderate-risk (Class B)	Remissive high-risk (Class C)	Progressive high-risk (Class D)
	OR (95% CI), P value	OR (95% CI), P value	OR (95% CI), P value
Age (per 5 years)	0.96 (0.95-0.98), <0.001	1.03 (1.02-1.04), <0.001	0.96 (0.95-0.98), <0.001
Gender			
Male	1.00 (Reference)	1.00 (Reference)	1.00 (Reference)
Female	0.99 (0.92-1.05), 0.659	0.96 (0.90-1.03), 0.276	0.92 (0.82-1.02), 0.124
BMI (per 5 kg/m²)	0.66 (0.62-0.70), <0.001	1.16 (1.10-1.22), <0.001	1.16 (1.05–1.22), <0.001
Hypertension			
No	1.00 (Reference)	1.00 (Reference)	1.00 (Reference)
Yes	0.99 (0.92-1.07), 0.794	1.05 (0.98-1.13), 0.174	1.00 (0.89-1.13), 0.948
Diabetes			
No	1.00 (Reference)	1.00 (Reference)	1.00 (Reference)
Yes	0.92 (0.83-1.03), 0.156	0.95 (0.85-1.06), 0.380	0.87 (0.72-1.05), 0.141

The Stable Low-Risk group (Class A) served as the reference category for all models. BMI, body mass index; OR, odds ratio; and CI, confidence interval.

### 3.3 Association of biological aging trajectories with incident urolithiasis

To examine how varying aging profiles influence urolithiasis development over time, we implemented Cox proportional hazards regression (**Table 3**). The unadjusted analysis (Model 1) revealed distinct risk profiles: the Progressive High-Risk group demonstrated a 21% increased vulnerability to lithogenesis (HR = 1.21; 95% CI, 1.00–1.45; P = 0.047), whereas the Remissive High-Risk group was associated with a significantly lower risk (HR = 0.81, 95% CI: 0.71–0.92, P = 0.002). Upon adjusting for the full spectrum of demographic and metabolic confounders (Model 3), the previously significant association within the Progressive High-Risk cohort underwent attenuation and lost statistical significance (HR = 1.14, 95% CI: 0.95–1.37, P = 0.170). In contrast, individuals in Remissive High-Risk group maintained a significantly lower risk of developing kidney stones (HR = 0.87, 95% CI: 0.76–0.99, P = 0.041).

**Table 3:** Association of longitudinal biological aging trajectories with the risk of incident urolithiasis.

Trajectories	Model 1	Model 2	Model 3
	HR (95%CI), P value	HR (95%CI), P value	HR (95%CI), P value
Stable Low-Risk (Class A)	1.00 (Reference)	1.00 (Reference)	1.00 (Reference)
Stable Moderate-Risk (Class B)	1.10 (0.97-1.24), 0.129	1.05 (0.93-1.19), 0.450	1.05 (0.93-1.19), 0.452
Remissive High-Risk (Class C)	0.81 (0.71-0.92), 0.002	0.87 (0.76-0.99), 0.041	0.87 (0.76-0.99), 0.041
Progressive High-Risk (Class D)	1.21 (1.00-1.45), 0.047	1.14 (0.95-1.37), 0.169	1.14 (0.95-1.37), 0.170

Model 1: unadjusted.

Model 2: age, gender, and BMI.

Model 3: age, gender, BMI, SBP, DBP, hypertension, and diabetes.

HR, hazard ratio; CI, confidence interval; BMI, body mass index; SBP, systolic blood pressure; DBP, diastolic blood pressure; HR, hazard ratio; and CI, confidence interval.

### 3.4 Mediation analysis of metabolic factors

**Figure 2:**
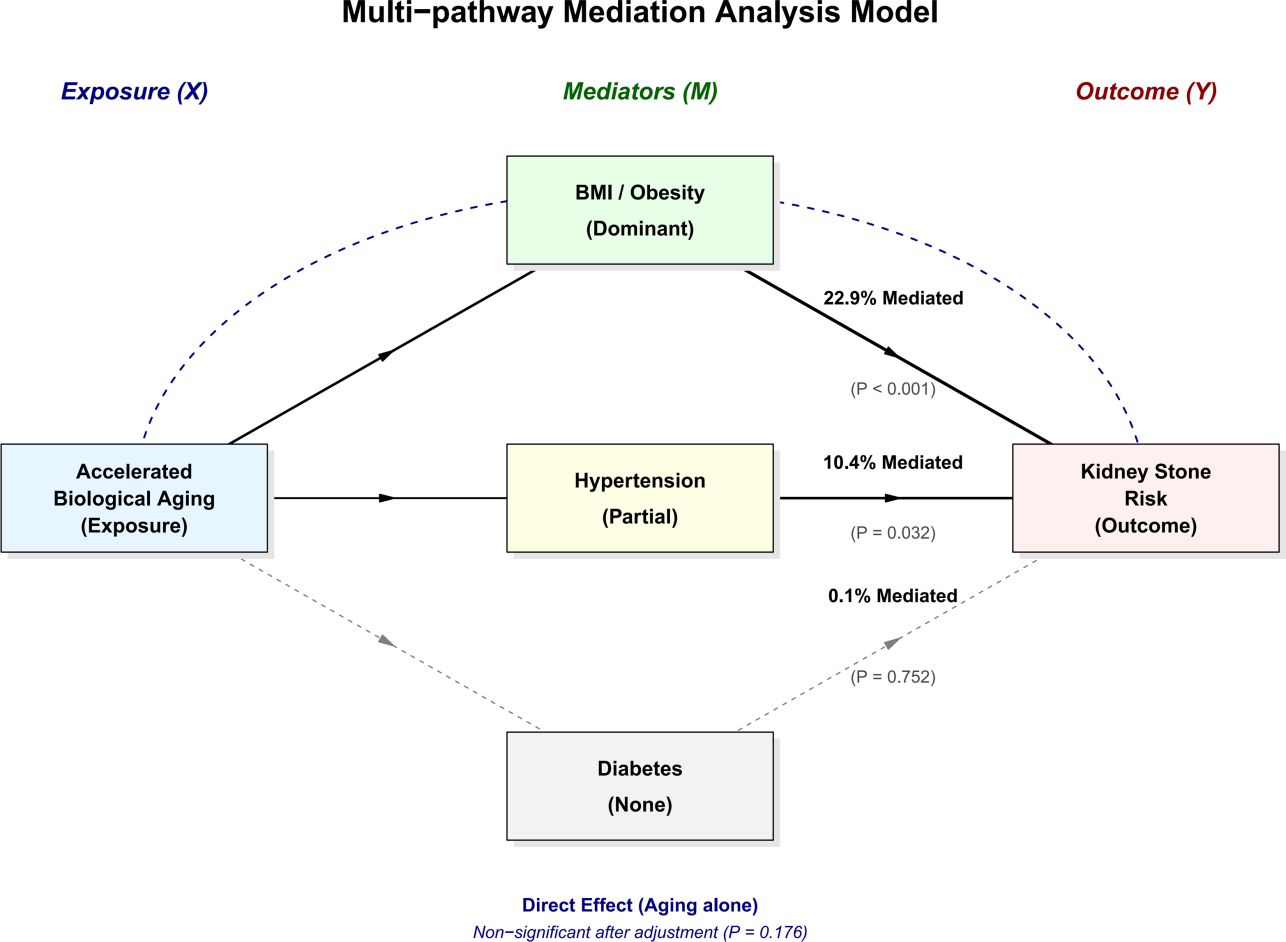
Causal mediation analysis of the association between biological aging trajectories and incident urolithiasis. The model illustrates the mediating roles of metabolic factors. BMI (22.9%, P < 0.001) and hypertension (10.4%, P = 0.032) significantly mediated the association, while diabetes showed no significant effect (P = 0.752). The direct effect of aging became non-significant (P = 0.176) after adjustment. Solid and dashed lines represent significant and non-significant pathways.

To directly test whether metabolic dysfunction drives lithogenesis, we performed a multi-pathway causal mediation analysis (**Figure 2**; **Supplementary Table 3**). Notably, the direct effect of the Progressive High-Risk trajectory on urolithiasis became non-significant after accounting for these mediators (P = 0.176), indicating full mediation. Mediational pathways revealed that BMI accounted for 22.9% of the total association between accelerated aging and urolithiasis risk (P < 0.001). Hypertension played a secondary yet significant role, mediating 10.4% of the effect (P = 0.032). In contrast, the mediating contribution of diabetes was negligible (0.1%, P = 0.752).

### 3.5 Sensitivity and subgroup analyses

To verify the stability of our estimates and mitigate potential reverse causality, we recalculated the models after omitting individuals with pre-existing diabetes or hypertension (**Supplementary Table 4**). In this restricted cohort, the predictive value of the biological aging trajectories remained robust. Notably, the Progressive High-Risk group maintained a significant independent association with incident urolithiasis (HR = 1.26, 95% CI: 1.00–1.58, P = 0.047), while the Remissive High-Risk group continued to confer significant protection (HR = 0.81, 95% CI: 0.68–0.96, P = 0.013).

Subgroup analyses stratified by age, gender, BMI, and comorbidities were further performed to explore potential effect modifiers (**Supplementary Table 5**). We observed no meaningful interactions across these strata (all P for interaction > 0.05), indicating a consistent association regardless of metabolic status. The lithogenic risk linked to progressive aging appeared more prominent among female participants (HR = 1.41; 95% CI, 1.02–1.95; P = 0.035). While this link did not reach the threshold for significance in the male subgroup (HR = 1.12; 95% CI, 0.89–1.40; P = 0.331), the formal test for sex-based interaction did not yield statistical significance (P = 0.256).

## 4 Discussion

This investigation offers a longitudinal perspective to systematically examine the dynamic association between biological aging trajectories and incident urolithiasis risk in a Chinese population. By applying unsupervised machine learning (kml3d) to longitudinal physiological data, we captured the heterogeneity of the aging process beyond static measurements (19). Our findings highlight two key points. First, the Remissive High-Risk trajectory was linked to a lower risk of lithogenesis (HR = 0.87; 95% CI: 0.76–0.99), suggesting an inherent capacity for physiological resilience (20). Second, while the Progressive High-Risk trajectory showed elevated risk in unadjusted analyses (HR = 1.21; 95% CI: 1.00–1.45), this excess risk diminished and became non-significant in fully adjusted models. This pattern matches our mediation analysis, showing that the risk in accelerated agers is driven largely by downstream metabolic factors, specifically BMI and hypertension, rather than aging itself.

Unlike cross-sectional studies that miss these dynamic changes (21), our trajectory analysis tracks how homeostatic dysregulation evolves. Identifying the Progressive High-Risk trajectory is clinically significant, as it signals early systemic physiological degradation (22). Our sensitivity analysis confirmed that accelerated aging predicts urolithiasis risk even in metabolically healthy individuals without diagnosed hypertension or diabetes. This fits the Geroscience hypothesis, which holds that biological aging drives molecular damage well before overt clinical comorbidities appear (23). Thus, the lithogenic process likely begins at the cellular level long before a stone is visible on imaging. This suggests that monitoring biological age could help shift focus from reactive treatment toward early, proactive prevention.

The clinical relevance of these findings is underscored by the evolving landscape of urolithiasis management. In recent years, surgical intervention has seen a significant paradigm shift, particularly in percutaneous surgery where technological advancements have redefined the efficiency of stone fragmentation and clearance (24). However, while these intraoperative optimizations address the immediate stone burden, they do not mitigate the underlying systemic predisposition to recurrence, which remains near 50% within five years if metabolic drivers are ignored (25). Our data complement this surgical evolution by proposing a parallel paradigm shift toward a biological “upstream” framework. By managing metabolic mediators, clinicians can effectively reduce the cumulative surgical burden, aligning proactive metabolic management with modern endourological progress (26).

Our causal mediation analysis directly tests the pathways linking aging to stone formation. We found that approximately one-third (33.3%) of the stone risk in the Progressive High-Risk group is collectively driven by BMI (22.9%) and hypertension (10.4%). Because the direct effect of aging was fully attenuated, metabolic decay appears to be a requisite intermediary. This is particularly relevant given China’s rapid urbanization, which has led to more metabolic disorders and a corresponding rise in stone burden (27, 28). To hypothetically explain the differing status of urolithiasis across our identified trajectory groups, we must examine the pathophysiology of these mediators. Mechanistically, the visceral adiposity associated with accelerated aging promotes insulin resistance and impairs renal ammoniagenesis, lowering urinary pH and increasing calcium excretion, creating a favorable environment for lithogenesis (29). Furthermore, the mediation effect of hypertension likely reflects aging-related microvascular damage and tubular stress, which reduces calcium reabsorption and promotes hypercalciuria (30). While hypertension mediated the risk, diabetes did not. This suggests that metabolic dysfunction driving stone formation may occur in the pre-diabetic phase or be driven by lipid toxicity itself (31). Consequently, the Progressive High-Risk group suffers the highest stone incidence due to the unchecked accumulation of these metabolic derangements, whereas the Remissive High-Risk group likely benefits from the restoration of metabolic homeostasis. These data provide a clear evidence-based roadmap: aggressive management of body weight and blood pressure can effectively decouple the link between systemic aging and local stone formation, shifting intervention from general lifestyle advice to a targeted mechanistic strategy.

Subgroup analyses showed gender-specific patterns. The link between accelerated aging and stone formation was strongest in women, matching recent trends in China where the gender gap in stone prevalence is narrowing (32). This likely reflects the loss of estrogen’s protective effect on metabolic health during aging, making women more susceptible to the stone-promoting effects of biological acceleration (33). In contrast, the clear protective benefit seen in the Remissive High-Risk trajectory, where biological age acceleration declined over time, supports the idea of reversibility (34). Aging is not fixed; interventions targeting metabolic health can slow the biological clock and reduce stone risk (35). The translational utility of our findings lies in the critical transition from reactive treatment toward precision proactive prevention (36). By integrating longitudinal biological age assessment into routine health screenings, healthcare providers can identify high-risk individuals in the Progressive phase long before stones become radiographically visible. In these cases, the 33.3% collective mediation effect of BMI and hypertension defines specific and modifiable therapeutic targets (25). We propose that monitoring the trajectory of biological aging, rather than relying on a single static measurement, represents a novel and dynamic biomarker for urolithiasis risk stratification (37). This approach facilitates the early implementation of intensive metabolic interventions, which potentially alters the patient’s physiological course and averts the onset of clinical stone disease (10).

This study derives significant methodological robustness from its longitudinal perspective, leveraging repeated assessments across a large-scale population of approximately 28, 000 participants. Using the unsupervised machine learning algorithm (kml3d), we captured the dynamic variation in biological aging. We also went beyond simple associations by adding multi-pathway mediation analysis. However, limitations exist. First, the reliance on B-mode ultrasonography for diagnosis may introduce misclassification bias. While non-contrast CT is the reference standard, ultrasound was utilized for its safety in longitudinal screening; its lower sensitivity for microlithiasis (< 2 mm) means our results likely represent a conservative estimate of the true association. Second, the reliance on a single-center health screening cohort in Yangzhou may introduce selection bias. Participants in routine health check-ups often exhibit higher health literacy and differing lifestyle patterns compared to the general population. Consequently, the generalizability of these findings to broader, ethnically or geographically diverse populations requires further validation through multi-center prospective studies. Additionally, specific dietary factors common in China, such as tea consumption and high sodium intake, were not fully recorded (38). Future studies using multi-omics data should identify the molecular markers specific to the Progressive High-Risk trajectory (39).

## 5 Conclusion

Distinct longitudinal trajectories of biological aging serve as robust early predictors of lithogenesis. The Progressive High-Risk phenotype increases stone risk, an effect fully mediated by metabolic factors, particularly BMI and hypertension. Conversely, the Remissive High-Risk trajectory shows that physiological dysregulation can reverse, and that this recovery provides significant protection. These findings suggest that targeting biological aging, specifically through weight management and blood pressure control, may offer a new, upstream strategy for preventing kidney stones.

## Data Availability

The raw data supporting the conclusions of this article will be made available by the authors, without undue reservation.
