# Supplementary material for: Longitudinal biological aging trajectories and incident urolithiasis: a causal mediation analysis role of metabolic dysfunction in a large Chinese cohort
Source: Front Endocrinol (Lausanne). 2026 Apr 17;17:1824716. doi: 10.3389/fendo.2026.1824716 (PMC13132696; doi:10.3389/fendo.2026.1824716)
Supplement: Supplementary file 1 [file DataSheet1.docx]

Supplementary Material

**Supplementary Figure 1** Flow diagram of the screening of participants.

**Supplementary Table 1** Model fit statistics for trajectory selection.

**Supplementary Table 2** Classification accuracy and distribution of trajectory groups (k=4).

**Supplementary Table 3** Causal mediation analysis of the association between the Progressive High-Risk aging trajectory and incident urolithiasis risk mediated by metabolic factors.

**Supplementary Table 4** Sensitivity analysis of the association between aging trajectories and urolithiasis in participants without hypertension or diabetes.

**Supplementary Table 5** Subgroup analysis of urolithiasis risk in the Progressive High-Risk trajectory.


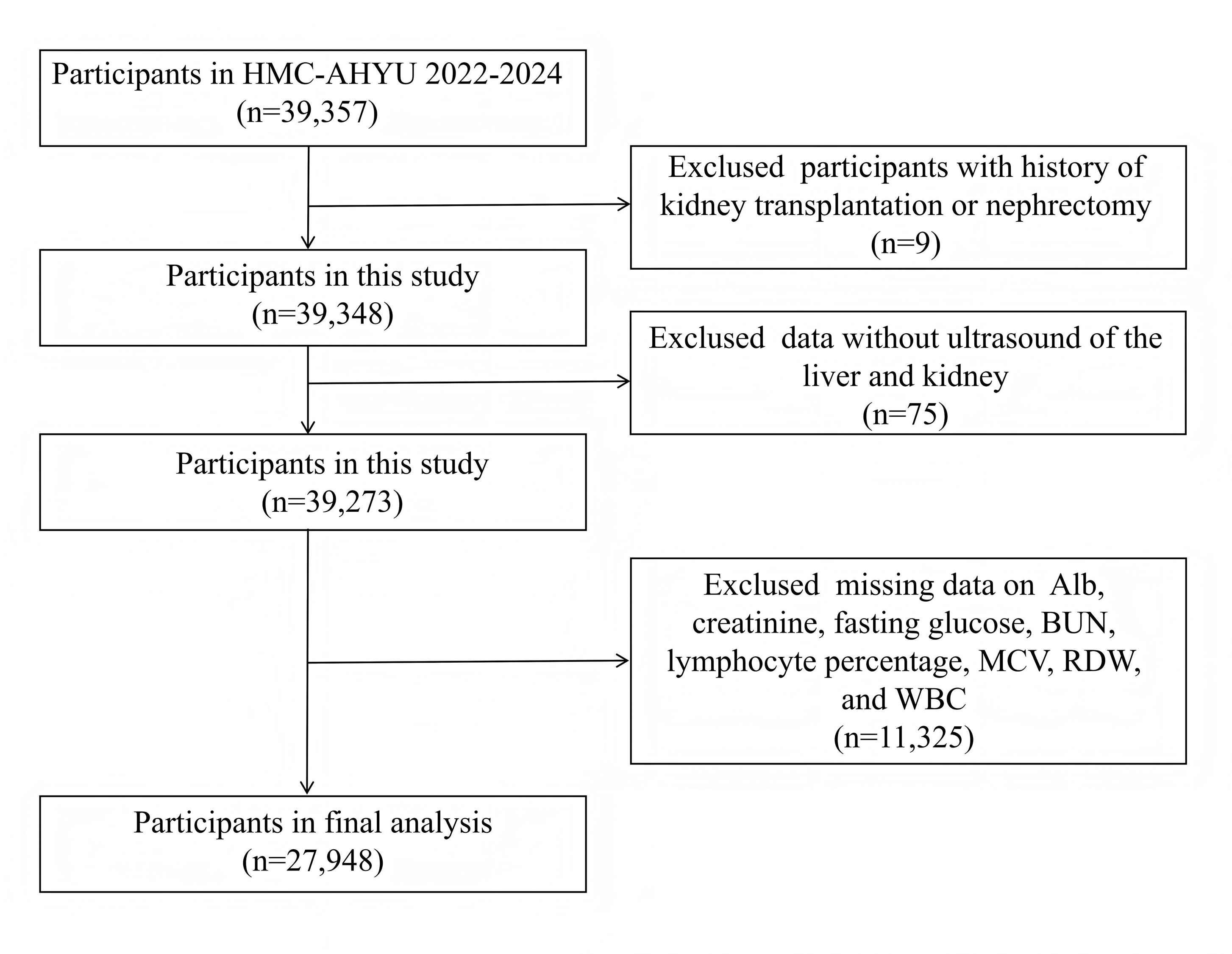


**Supplementary Figure 1** Flow diagram of the screening of participants.

HMC-AHYU, Affiliated Hospital of Yangzhou University; Alb, Albumin; BUN, blood urea nitrogen; MCV, mean cell volume; RDW, red cell distribution width; WBC, white blood cell count.

**Supplementary Table 1** Model fit statistics for trajectory selection

| **Clusters (k)** | **AIC** | **BIC** | **CH** |
| --- | --- | --- | --- |
| 2 | 57027.08 | 57043.56 | 11356.74 |
| 3 | 51030.42 | 51055.13 | 11105.68 |
| 4 | 48799.44 | 48832.39 | 10262.08 |
| 5 | 40953.68 | 40994.87 | 9598.00 |
| 6 | 36754.18 | 36803.61 | 8968.28 |

AIC, Akaike Information Criterion; BIC,Bayesian Information Criterion；**CH，Calinski-Harabasz.**

**Supplementary Table 2** Classification accuracy and distribution of trajectory groups (k=4)

| **Trajectory Group** | **Sample Size (n)** | **Proportion (%)** | **AvePP** |
| --- | --- | --- | --- |
| Stable Low-Risk (Class A) | 7,633 | 27.31% | 0.925 |
| Stable Moderate-Risk (Class B) | 9,687 | 34.66% | 0.944 |
| Remissive High-Risk (Class C) | 8,607 | 30.80% | 0.914 |
| Progressive High-Risk (Class D) | 2,021 | 7.23% | 0.938 |
| Overall | 27,948 | 100.0% | 0.930 |

AvePP，Average Posterior Probability.

**Supplementary Table 3** Mediation analysis of the association between the Progressive High-Risk aging trajectory and incident urolithiasis risk mediated by metabolic factors.

| **Mediator** | **Total Effect ^a^** | **Direct Effect (ADE)** | **Indirect Effect (ACME)** | **Proportion Mediated (%)** |
| --- | --- | --- | --- | --- |
|  | **Estimate, P value** | **Estimate, P value** | **Estimate, P value** |  |
| BMI | 0.0118 (P = 0.064) | 0.0088 (P = 0.176) | 0.0027 (P < 0.001) | 22.9% |
| Hypertension | 0.0122 (P = 0.040) | 0.0107 (P = 0.076) | 0.0013 (P = 0.032) | 10.4% |
| Diabetes | 0.0121 (P = 0.052) | 0.0121 (P = 0.048) | 0.0000 (P = 0.752) | 0.1% |

BMI, body mass index; ACME, average causal mediation effect; ADE, average direct effect; ^a^ The total effect represents the combined pathway from the trajectory to urolithiasis. Estimates are presented as regression coefficients on the probability scale. Bold values indicate statistical significance (P < 0.05).

**Supplementary Table 4** Sensitivity analysis of the association between aging trajectories and urolithiasis in participants without hypertension or diabetes.

| **Trajectory Group** | **HR (95% CI)** | **P Value** |
| --- | --- | --- |
| Stable Low-Risk (Class A) | 1.00 (Reference) | - |
| Stable Moderate-Risk (Class B) | 1.07 (0.91–1.25) | 0.415 |
| Remissive High-Risk (Class C) | 0.81 (0.68–0.96) | 0.013 |
| Progressive High-Risk (Class D) | 1.26 (1.00–1.58) | 0.047 |

Analysis excluded participants with baseline hypertension or diabetes to minimize reverse causality and metabolic confounding. Models were adjusted for age and gender.

**Supplementary Table 5** Subgroup analysis of urolithiasis risk in the Progressive High-Risk trajectory.

| **Subgroup** | **HR (95% CI)** | **P Value** | **P for Interaction** |
| --- | --- | --- | --- |
| Gender |  |  | 0.256 |
| Male | 1.12 (0.89–1.40) | 0.331 |  |
| Female | 1.41 (1.02–1.95) | 0.035 |  |
| Age |  |  | 0.608 |
| < 40 years | 1.36 (0.97–1.91) | 0.076 |  |
| 40–60 years | 1.22 (0.95–1.58) | 0.125 |  |
| ≥60 years | 0.97 (0.61–1.52) | 0.878 |  |
| BMI |  |  | 0.901 |
| < 25 kg/m² | 1.16 (0.87–1.53) | 0.310 |  |
| 25–30 kg/m² | 1.19 (0.90–1.56) | 0.223 |  |
| ≥30 kg/m² | 1.06 (0.60–1.88) | 0.832 |  |
| Hypertension |  |  | 0.875 |
| No | 1.12 (0.87–1.45) | 0.373 |  |
| Yes | 1.17 (0.89–1.54) | 0.265 |  |
| Diabetes |  |  | 0.670 |
| No | 1.19 (0.98–1.44) | 0.074 |  |
| Yes | 1.42 (0.69–2.90) | 0.337 |  |

HRs were calculated using Model 3, adjusting for all baseline covariates (age, gender, BMI, systolic blood pressure, diastolic blood pressure, hypertension, and diabetes) except for the stratification variable itself. HR, hazard ratio; CI, confidence interval; BMI, body mass index.
